# Dosimetric comparison between brachytherapy and MR-Linac as a boost modality for locally advanced cervical cancer

**DOI:** 10.1016/j.ctro.2025.101098

**Published:** 2025-12-17

**Authors:** Renske van Noortwijk, Petra S. Kroon, Katelijne M. van Vliet-van den Ende, Erik H. Brondijk, Gonda G. Sikkes, Alexis N.T.J. Kotte, Ina M. Jürgenliemk-Schulz, Femke van der Leij, Astrid L.H.M.W. van Lier

**Affiliations:** Department of Radiotherapy, University Medical Center Utrecht, Heidelberglaan 100, 3584CX Utrecht, Netherlands

## Abstract

**Background and purpose:**

Standard treatment for locally advanced cervical cancer (LACC) is chemoradiotherapy followed by a brachytherapy (BT) boost. However, BT is not always feasible and magnetic resonance (MR)-guided adaptive radiotherapy on the MR-Linac (MRL) might be an alternative. To investigate the dosimetric feasibility of MRL, BT and MRL treatment plans were compared intra-patient in terms of dosimetric differences, next to anatomical and conformity variations.

**Materials and methods:**

Two groups of ten patients with LACC treated with BT boost were selected: group 1 included patients for which at least one clinically established (EMBRACE II) treatment planning constraint was not achieved during BT, in group 2 all planning constraints were achieved.

BT treatment plans were compared with MRL treatment plans (based on MRI scans without applicator in place) intra-patient, in terms of dose-volume histogram (DVH) parameters, target-to-OAR (organ at risk) surface distances and conformity ratios.

**Results:**

Group 1 resulted in similar prescribed target dose levels for MRL compared to BT, for group 2 all prescribed target dose levels were significantly higher for BT. Rectum D2cm^3^ was higher for all MRL treatment plans. Volumes of higher dose levels were larger for BT, volumes of lower dose levels were larger for MRL and the CTV_HR_ to OAR (rectum, sigmoid, bowel) surface distance was greater for BT.

**Conclusion:**

This retrospective study demonstrates that with an MRL boost plan, in some situations it is possible to achieve established planning constraints. However, as rectum doses are higher and dose distributions are fundamentally different, BT remains the modality of choice. Clinical trials are necessary to investigate the influence of the MRL dose distribution on oncological outcomes.

## Highlights

• BT and MRL treatment plans were compared intra-patient, within two patient groups.
• The dosimetric performance of MRL with respect to BT differed between groups.
• High dose-volumes are larger for BT, low dose-volumes are larger for MRL.
• Distance of CTV_HR_ to rectum, sigmoid and bowel surface increases with applicator in-situ.
• The influence of MRL dose distributions on clinical outcomes must be investigated.

## Introduction

Cervical cancer is one of the most common cancer types among women; in The Netherlands, 5 in 100.000 women were diagnosed with cervical cancer in 2022 [1]. Standard treatment for locally advanced cervical cancer (LACC) is external beam radiotherapy (EBRT) with concomitant chemotherapy, followed by a brachytherapy (BT) boost. Brachytherapy is proven to be important for optimal local control and cure [2]. The EMBRACE II study, a multicentre prospective international cohort study investigating magnetic resonance imaging (MRI)-based image-guided adaptive brachytherapy (IGABT) in the treatment of cervical cancer, resulted in a 5-year overall local control rate of 92 % and a 5-year disease-free survival of 73 % [3,4].

Nevertheless, brachytherapy as a boost after chemoradiation is invasive and thereby not always a feasible treatment option. In some cases, patients are unable to endure applicator insertion due to physical limitations (e.g., large tumour volumes, not fit enough to undergo treatment, use of therapeutic anticoagulants), psychological reasons, or other contra indications [5]. In these cases, an alternative option for a boost would be convenient. In previous studies, patients have been treated with stereotactic body radiotherapy (SBRT) on a conventional LINAC (linear accelerator) or CyberKnife [6,7]. During these treatments, high radiation doses (13–40 Gy delivered in 2–5 fractions as summarized by Miranda et al. [5]) were delivered to the tumour, aiming to approach the brachytherapy dose levels. Results of these treatments with SBRT show promising target-related outcomes, highlighting the potential for SBRT to be an alternative when BT is not feasible. However, high toxicity is an issue, which caused an early suspension for a Phase II trial on SBRT boost for LACC on a conventional LINAC [8,9]. This trial was limited to a relatively high median tumour volume (median clinical target volume (CTV) of 82 cm^3^ (30 – 165 cm^3^). Next to this, tumour dose aims were prioritized over organ-at-risk (OAR) dose aims enlarging the risk for high doses outside the target as a conventional LINAC with cone-beam CT (computed tomography) imaging setting was used.

An option to answer the need for more precise external beam radiation delivery is MR-guided adaptive radiotherapy on the MR-Linac (MRL) (Elekta AB, Stockholm, Sweden): a linear accelerator system integrated with an MRI scanner [10,11]. Treatment plans can be optimized regarding the daily anatomy of the patient; plans can be altered based on position, adapt-to-position (ATP), or the shape of the target, adapt-to-shape (ATS) [11]. This method is already being used for other tumour sites (e.g., prostate, rectum and more tumour sites), but it has not yet been clinically approved as a boost modality for cervical cancer [[12], [13], [14]]. An ongoing clinical trial (MARGARITA study, clinicaltrials.gov identifier: NCT05937958) investigates the clinical outcomes of MRL as a boost modality for cervical cancer, for patients not eligible for brachytherapy [15]. To interpret these outcomes with respect to BT, it is interesting to get a closer look on the dosimetric differences between BT and MRL, to gain more knowledge on the dosimetric distributions and their influence on target and OAR dose.

Therefore, to investigate the dosimetric feasibility of MRL as a boost modality for cervical cancer if BT is not possible, we compared MRL boost plans with the standard of care BT boost plans. We determined the dosimetric difference between these two modalities, as well as anatomical and conformality variations. For this planning study, a unique dataset with MRI scans with and without brachytherapy applicator (and needles) scanned at the same day was used.

## Materials and methods

Patient selection

In this study, imaging data of twenty patients with LACC treated at our institution between August 2016 and February 2024 with chemoradiation followed by a BT boost was investigated. The use of these imaging datasets was approved by the local ethics committee (Institutional Review Board approval: WAG/mb/20/500028).

Two patient groups were set up; group 1 consisted of patients for whom not every BT treatment planning constraint was achieved (based on EMBRACE II treatment planning hard and soft constraints [3]). Included were ten patients that met one or more of the selection criteria as presented in Supplementary material 1. In group 2, the ten most recently treated patients, for whom all BT treatment planning constraints were achieved, were included.

BT treatment planning

In our institution, LACC patients are treated according to the EMBRACE II protocol [3]. The treatment consists of EBRT with VMAT (Volumetric Modulated Arc Therapy), administering an elective dose of 45 Gy (25 fractions of 1.8 Gy) and a simultaneous integrated boost (SIB) to a total dose of 55.0 Gy or 57.5 Gy to pathological lymph nodes, in combination with weekly chemotherapy (cisplatin 40 mg/m^2^). Thereafter, a high-dose-rate (HDR) BT boost is applied, which consists of two applications with a 1-week interval, comprising two fractions (four 7 Gy fractions in total, to a total physical dose of 28 Gy). The BT treatment workflow is explained in detail by Van Vliet-Van den Ende et al. (2024) [16].

Two treatment plans are used for BT: one for fractions 1 and 2 and one for fractions 3 and 4. Before each fraction, an MRI (pre-radiation) scan is performed to verify whether the anatomy is still representative with respect to the treatment plan. In a clinical setting, a urinary catheter is used to regulate the bladder filling in order to balance the dose in the bowel and the bladder [16].

After fractions 1 and 2, the delivered dose is determined based on the two pre-radiation scans, allowing the treatment plan for the remaining two fractions to account for the delivered dose in fraction 1 and 2. Consequently, the pre-radiation scans of fractions 3 and 4 can be used to calculate the delivered dose in these fractions. In this study, the delivered dose for fractions 1 and 2 and the prescribed dose for fractions 3 and 4 were used.

Treatment planning was performed in Oncentra Brachy, version 4.6 (Elekta AB, Stockholm, Sweden), based on a Flexitron afterloader (Elekta, Veenendaal, The Netherlands). Total dose used for calculation of dose parameters includes a homogeneous dose of 25 x 1.8 Gy during the EBRT phase (44.3 Gy EQD2 for α/β = 10, and 43.2 Gy EQD2 for α/β = 3) and the final BT boost dose.

For group 1, BT plans were optimized based on the OAR planning hard constraints being dose limiting (isotoxic treatment planning), to ensure that each plan was optimized using the same priorities. The applicator was excluded (except tandem part) from the target delineations in the BT treatment plans.

MRL treatment planning

Within the MARGARITA study (NCT05937958), MRL boost treatment plans are performed in six fractions, based on a sub-fractionation workflow [15,17]. During every fraction, the treatment plan can be fully adapted to the daily anatomy using the ATS approach and motion management. The physical boost planning dose constraints result from the EMBRACE II planning constraints in EQD2, after subtracting the EBRT dose of 25 x 1.8 Gy (EQD2 44.3 for α/β = 10, see Supplementary material 1) and recalculating for six fractions.

For this planning study, the MRI scan acquired before the first BT application (i.e., without applicator) was used for treatment planning of the MRL boost (‘MRpreApp’ in Van Vliet-Van den Ende et al. [16]) assuming six identical fractions. Gross tumour volume (GTV), high-risk clinical target volume (CTV_HR_) and intermediate-risk clinical target volume (CTV_IR_) were delineated. A treatment plan was created in Monaco, version 6.2.1.0 (Elekta AB, Stockholm, Sweden), considering the EMBRACE II planning constraints, with OAR planning hard constraints being the dose limiting factor. A PTV-margin of 3 mm was added to CTV_HR_ and GTV (according to MARGARITA study protocol), the doses in these PTVs were used to assess the CTV_HR_ and GTV with respect to dose constraints. Total dose used for calculation of dose parameters include the same EBRT dose as used for the BT treatment plans.

DVH parameters

For each patient, target ((PTV-)CTV_HR_ D90%, (PTV-)CTV_HR_ D98%, (PTV-)GTV D98%, CTV_IR_ D98%) and OAR (D2cm^3^ for bladder, sigmoid, rectum and bowel) prescribed dose levels were calculated in EQD2 using the simple calculation method (target: α/β = 10, OAR: α/β = 3) [18].

Next to this, a range of additional DVH parameters between V50% and V150% for target tissue (α/β = 10) and between V25% and V90% for normal tissue (α/β = 3) was calculated (see Supplementary material 2). These parameters were based on V100% for BT being the volume receiving the prescription dose (7 Gy per fraction). For BT treatment plans, the parameters were determined in Oncentra Brachy using the ‘ROI from Isoline’ option in Brachy Planning activity. As two treatment plans per patient were used, the average of these two plans was used for analysis. For MRL treatment plans, the corresponding BT EQD2 dose for each parameter was used to recalculate the physical boost for six fractions after subtracting the EBRT EQD2 dose.

Analysis of anatomy

To investigate the anatomical differences between BT and MRL in-silico (with and without applicator in place), a measure was developed for the distance between target and OAR surfaces. Using an in-house developed software tool (Volumetool [19]), the distance was determined between each individual point on the CTV_HR_ surface and its corresponding closest point on each OAR surface (bladder, rectum, sigmoid, bowel). In this tool, each VOI was triangulated and the distance was calculated between the centres of triangles. This resulted in a set of distances coupled to each OAR, of which the 5th percentile was calculated that was marked as the shortest distance between the target and OAR.

Conformity analysis

The conformity of BT and MRL treatment plans was assessed using indices derived from definitions according to literature [[20], [21], [22]]. Conformity index (CI) (1) and healthy tissues conformity index (HTCI) (2) were calculated (both range from 0 to 1):

(1) $$\mathrm{CI}={\mathrm{TV}}_{\mathrm{PIV}}/TV$$

(2) $$\mathrm{HTCI}={\mathrm{TV}}_{\mathrm{PIV}}/PIV$$

where TV represents target volume (PTV-CTV_HR_ for MRL and CTV_HR_ for BT, or CTV_IR_ for both modalities), TV_PIV_ represents the volume of TV receiving the prescription isodose (PIV). All prescription isodoses were based on the EMBRACE II soft planning dose constraints (see Supplementary material 1). HTCI indicates the amount of prescription dose outside the target, CI indicates the coverage of the target by the prescription dose. To illustrate, for CTV_HR_ a CI of ≥ 0.9 shows that D90% ≥ 90 Gy was reached, any value above 0.9 indicates that a larger portion of the target than minimally required receives the prescription dose.

Statistical tests

All statistical tests were performed in GraphPad Prism v8.0.2 using the paired Wilcoxon signed-rank test, considering a p-value < 0.05 to be significant.

## Results

Tumour and application characteristics for group 1 and 2 are summarized in Table 1. Median CTV_HR_ volumes for group 1 and group 2 were 53 cm^3^ (range: 25 – 90 cm^3^) and 20 cm^3^ (range: 16 – 26 cm^3^), respectively. Considering all 20 patients, median bladder volume was 126 cm^3^ (range: 35 – 240 cm^3^) within BT treatment plans and 64 cm^3^ (range: 41 – 362 cm^3^) within MRL treatment plans. The most prevalent applicator type in both groups was Venezia (Elekta Brachytherapy, The Netherlands) and the median number of interstitial needles used per application was five (range: 3 – 8) and zero (range: 0 – 2) for group 1 and 2, respectively.

**Table 1:** Tumour and application characteristics. Target volumes are based on the planning MRI of the first BT fraction, bladder volumes are specified for BT and MRL planning MRI scans separately.

	**All (n = 20)**	**Group 1 (n = 10)**	**Group 2 (n = 10)**
Median GTV volume (cm^3^) (range)	7 (1–65)	19 (6–65)	3 (1–6)
Median CTV_HR_ volume (cm^3^) (range)	25 (16–90)	53 (25–90)	20 (16–26)
Median CTV_IR_ volume (cm^3^) (range)	67 (42–175)	119 (67–175)	55 (42–67)
Median bladder volume – BT (cm^3^) (range)	126 (36–249)	111 (51–249)	138 (36–232)
Median bladder volume – MRL (cm^3^) (range)	64 (41–362)	72 (44–362)	58 (41–362)
Applicator type			
*Venezia (Elekta)*	12 (60 %)	7 (70 %)	5 (50 %)
*Geneva (Elekta)*	6 (30 %)	1 (10 %)	5 (50 %)
*Utrecht (Elekta)*	2 (10 %)	2(20 %)	−
Median # of needles (range)			
*Right*	1 (0–4)	2 (0–4)	0 (0–2)
*Left*	2 (0–6)	2 (0–6)	0 (0–2)
*Total*	3 (0–8)	5 (3–8)	0 (0–2)

**Fig. 1:**
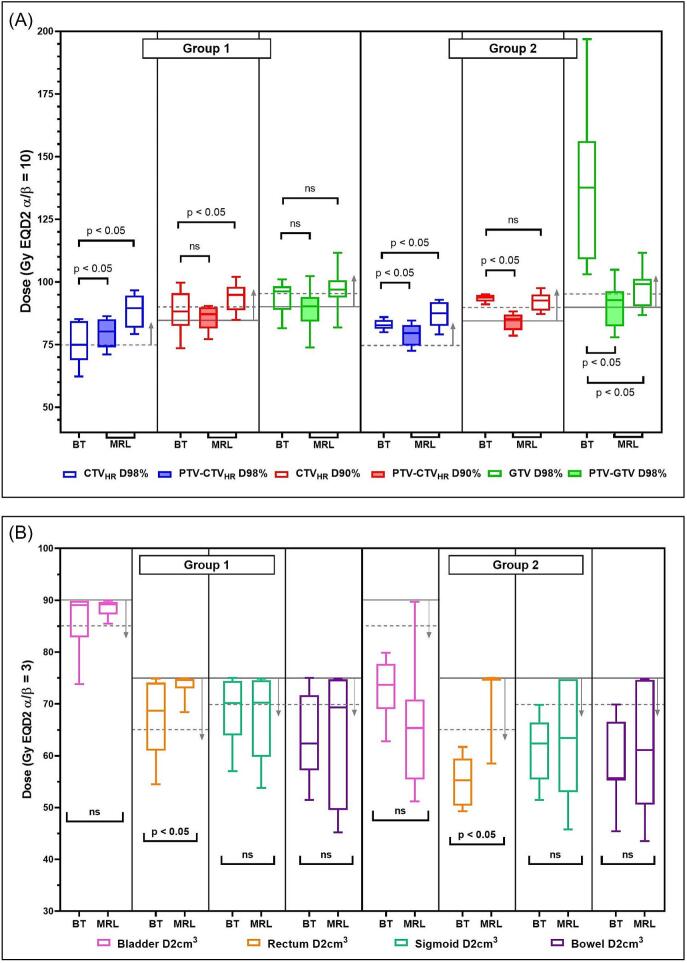
(A) prescribed target dose levels (EQD2 α/β = 10) for BT (CTV_HR_) and MRL (PTV-CTV_HR_ and CTV_HR_) and (B) prescribed OAR dose levels for BT and MRL. Grey lines indicate hard constraints, dashed grey lines indicate soft constraints. The direction of the arrow indicates whether the constraint is maximum (down) or minimum (up). Statistical significance is indicated per comparison (p < 0.05 = significant, ns = not significant).

In Fig. 1, prescribed dose levels for target and OAR are displayed for both groups separately. Within group 1, prescribed target dose levels were not significantly different between BT and MRL, with the exception of CTV_HR_ D98% (p-value 0.037). (PTV-)CTV_HR_ D90% hard constraint (>85 Gy) was not achieved within three patients for both BT and MRL (patients vary). Within group 2, all prescribed target dose levels were significantly higher for BT; CTV_HR_ D90% > 85 Gy was achieved in all patients, while for MRL this hard constraint was not achieved for five patients. All OAR prescribed dose levels did not exceed hard constraints, rectum D2cm^3^ doses were significantly higher for MRL.

**Fig. 2:**
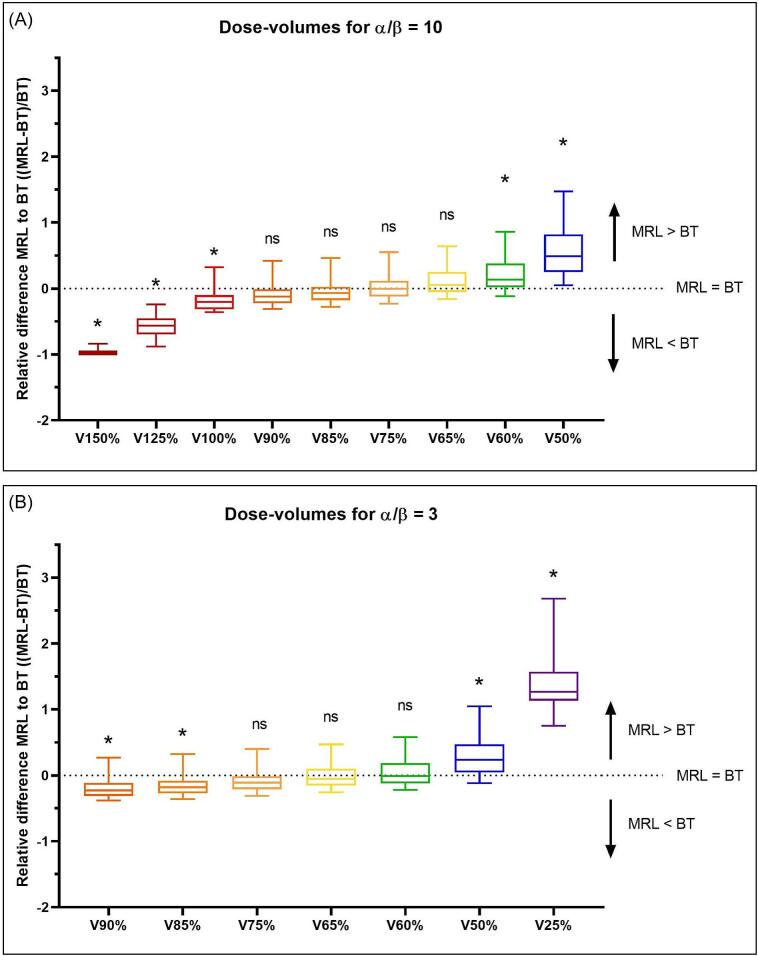
Relative difference of dose-volume parameters for (a) target tissue (α/β = 10) and (B) normal tissue (α/β = 3). V100% corresponds with the volume receiving the prescription dose (7 Gy per fraction). Relative difference was calculated with respect to BT ((MRL-BT)/BT), a value of zero indicates no difference between MRL and BT. A value above zero states the MRL treatment plan results in a higher dose volume, a value below zero states a higher dose volume within the BT treatment plan. Statistical significance (p-value < 0.05) is indicated (* = significant, ns = not significant).

In Fig. 2, two ranges of dose volumes are shown. For target tissue, no significant difference between BT and MRL was observed between V90% and V65%. Higher dose-volumes were larger for BT, lower dose-volumes were larger within the MRL treatment plans. This trend was also visible for normal tissue: no significant difference was noted between V75% and V60%.

**Fig. 3:**
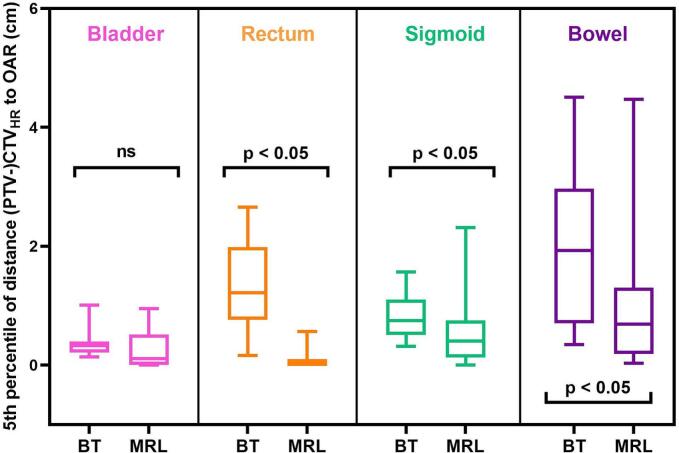
Shortest distances between target (ctv_HR_ for BT and PTV-CTV_HR_ for MRL) and OARs for BT and MRL, both groups combined. Statistical significance is indicated (p < 0.05 = significant, ns = not significant).

The shortest distance between target (CTV_HR_ for BT and PTV-CTV_HR_ for MRL) and OAR surfaces was determined, results for both group 1 and 2 combined are shown in Fig. 3. For rectum, sigmoid and bowel, the distance for MRL was significantly smaller compared to BT.

**Fig. 4:**
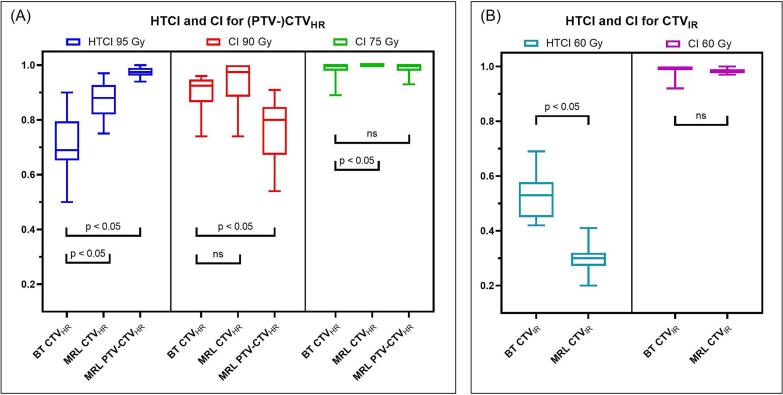
Results of conformity analysis.Healthy tissues conformity index (HTCI) and conformity index (CI) for BT and MRL treatment plans (A) CTV_HR_ (CTV_HR_ for BT, PTV-CTV_HR_ and CTV_HR_ for MRL) and (B) CTV_IR_. Statistical significance is indicated (p < 0.05 = significant, ns = not significant).

In terms of conformity of the treatment plans (Fig. 4), HTCI 95 Gy was lower for BT compared to MRL. On the contrary, the HTCI 60 Gy for CTV_IR_ was lower for MRL. Regarding the CI, only CI 90 Gy was significantly different: MRL plans resulted in a lower CI 90 Gy for CTV_HR_. The conformity ratios are displayed for both groups combined, as results for two groups separately were similar.

**Fig. 5:**
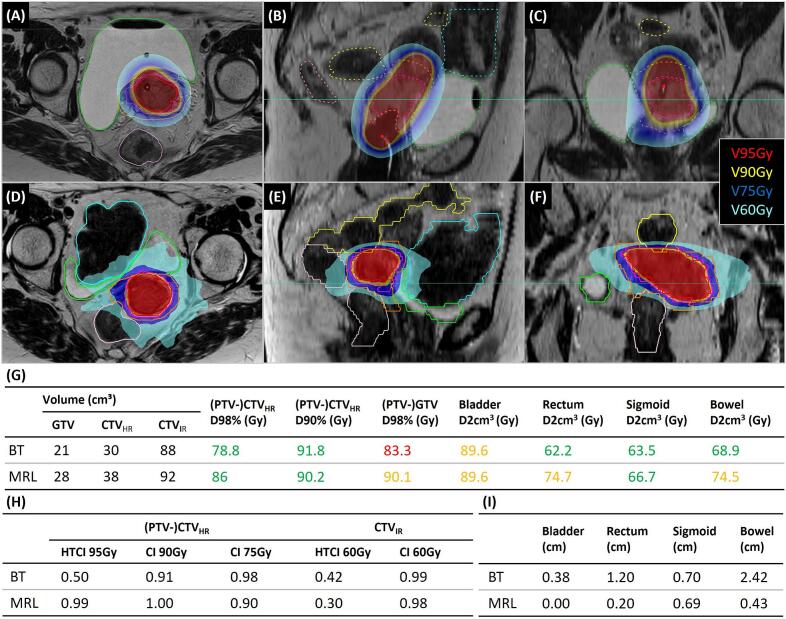
Example of a patient from group 1. Transversal, sagittal and coronal views of dose distribution for (A-C) BT treatment plans and (D-F) MRL treatment plans for the same patient. Planning constraint isodoses (V60 Gy, V75Gy, etc.)(EQD2 α/β = 10) corresponding to coloured areas are indicated. GTV (red), CTV_HR_ (magenta), CTV_IR_ (orange), bladder (green), rectum (light pink), bowel (blue) and sigmoid (yellow) are shown. Green lines in the sagittal and coronal view indicate the position of the corresponding transversal slide. Additionally, (G) target and OAR prescribed doses (green indicates achievement of soft constraint, orange indicates no achievement of soft constraint (hard constraint achieved if applicable), red indicates no achievement of hard constraint) are included, next to (H) HTCI and CI and (I) target-OAR distances.

In Fig. 5, dose distributions and (dose) volume metrics for both modalities are shown for a patient included in group 1 (see Supplementary material 3 for a group 2 patient example). The GTV D98% planning hard constraint (>90 Gy) was not achieved within the BT treatment plan, PTV-GTV D98% of the MRL treatment plan was 90.1 Gy. In both treatment plans, the bladder was dose-limiting as D2cm^3^ values were close to the planning hard constraint (<90 Gy). Only within the MRL treatment plan, rectum and bowel were also dose-limiting. The HTCI for CTV_IR_ was lower for MRL, while for CTV_HR_ the HTCI was higher for MRL. In terms of CI, all values were acceptable. Additionally, it has to be noted that all target-to-OAR distances were larger for BT.

## Discussion

This study provides insight into the dosimetric differences between BT and MRL as a boost modality for locally advanced cervical cancer, by comparing BT and MRL treatment plans for individual patients within two different groups. Our results show that for tumour cases where BT resulted in achieving all clinically established planning constraints. Within group 2, MRL resulted in significantly lower prescribed target doses and notably more patients did not achieve the (PTV-)CTV_HR_ D90% hard constraint (five for MRL vs. zero for BT). On the other hand, within group 1 prescribed target dose levels were not significantly different between BT and MRL, and the number of patients where the (PTV-)CTV_HR_ D90% planning constraint was not achieved was comparable (three patients).

The difference between group 1 and group 2is suspected to be highly influenced by a difference in tumour volume, which was 53 cm^3^ (median) for group 1 and 20 cm^3^ (median) for group 2. The results for group 1 underline the potential for a dosimetrically feasible MRL treatment when BT constraints are difficult to achieve, as MRL seems not to under-perform with respect to BT in terms of achievement of constraints. Next to this it has to be noted that, even though BT resulted in significantly higher prescribed target doses in group 2, in some cases planning constraints were achieved using MRL treatment planning as well.

For both modalities, OAR hard constraints were not exceeded and OAR D2cm^3^ prescribed doses were not significantly different for bladder, sigmoid and bowel. However, rectum D2cm^3^ prescribed doses were significantly lower for BT; in group 2 all BT rectum doses did not exceed 65 Gy (soft constraint) in contrast to MRL. This is an important distinguishment between BT and MRL treatment plans, as Mazeron et al. (2016) showed that a rectum D2cm^3^ < 65 Gy is related to more minor and less frequent rectal morbidity [23]. The rectal dose difference can likely be explained by the results from the distance analysis, which showed that the distance between CTV_HR_ and rectum, sigmoid and bowel surfaces was significantly larger in the BT situation compared to MRL. Induced by the applicator placement including tamponade, more distance can be created which allows more room in balancing target and OAR planning constraints. To increase the target-to-OAR surface distance in the MRL situation to potentially decrease OAR dose, the option to create more space by the use of a vaginal gel could be explored [24].

As MR-linac based SBRT as a boost modality for locally advanced cervical cancer is a novel approach, limited data are published regarding this topic. Hadi et al. (2022) investigated the feasibility and safety of an MR-guided SBRT boost following EBRT for ten patients who were not suitable for BT [25]. A ViewRay MR-linac system was used, and a median total dose of 21.0 Gy (range: 4.0–7.0 Gy) was administered in four (median) fractions. There was complete response and local control in six patients and nine patients (respectively) after a median follow-up of nine months and the treatment was well-tolerated in terms of toxicity. Although these results demonstrate potential feasibility of an MR-guided SBRT boost, the study population is small and the follow-up is too short for definite conclusions in terms of tumour control and morbidity. Therefore, taking into account the numerous clinical results for BT with higher local control based on more comprehensive data, the authors do emphasize that in their opinion BT remains standard of care [4,26,27].

Dincer et al. (2024) compared MR-guided SBRT and BT boost treatment plans for four patients previously treated with HDR BT [8]. Outcome measures were target doses and OAR doses; mean EQD2 D90% of CTV_HR_ for BT and PTV-CTV_HR_ for MR-guided SBRT were 89.7 Gy and 82.9 Gy, respectively. Mean CTV_HR_ volume was 21 cm^3^, which is comparable to the median volume for group 2 in our study (20 cm^3^). BT CTV_HR_ D90% doses were significantly higher, which is also in line with Dincer et al. (2024). However, results from group 1 of our study showed no significant difference between BT and MRL in terms of prescribed dose values. This highlights the value of taking the dosimetric planning performance of BT (which relates to tumour volume) into account when comparing dosimetric results.

In our study the difference between BT and MRL dose distributions was also demonstrated in terms of additional DVH parameters (V25%, V50%, etc.) and a conformity analysis (HTCI and CI). Using BT as a boost modality, the volume receiving high doses (V150%, V125% and V100% for tumour tissue) was larger, as a result of the steep dose fall-off and interstitial dose delivery. Using MRL as a boost modality, the volumes receiving low doses (V50% and V25%) were larger. This illustrates the fundamental dose distribution difference between BT and MRL, which was also reflected in the conformity analysis. CTV_IR_ HTCI 60 Gy was lower for MRL (more prescription dose outside target), as the area receiving a lower dose value is larger for MRL. The area receiving a higher dose was larger for BT, which partly explains the lower CTV_HR_ HTCI 95 Gy for BT. Another explanation for this is related to the treatment planning guidelines in the EMBRACE II protocol [3]. Herein it is stated that, during treatment planning, a margin of 1 cm should be applied cranial to the CTV_HR_ to account for geometric uncertainties in applicator positioning, which means the HTCI will always be closer to zero when this is implemented. Therefore, it has to be noted that the HTCI is a way to describe the dose distributions with respect to the target, but no definite conclusions should be drawn based only on this parameter.

Additionally, as this is a planning study, the influence of the different dose distributions on clinical results is unknown, which is a limitation of this study. While treatment plan results were similar in terms of prescribed target dose levels for group 1, it is not clear whether the high doses accomplished using a BT dose distribution are essential for tumour control. Conversely, it is not clear whether the larger low-dose volumes within MRL may have a negative influence on toxicity.

Next to this, this planning study was based on a relatively low number of patients. Therefore, it is necessary to perform clinical studies on using MRL as a boost modality for LACC on more patients in the future, to determine whether comparable tumour control can be established using a different dose distribution (e.g., MARGARITA study, NCT05937958) [15].

Another limitation is the retrospective nature of this study; as we used data of patients already treated with BT, only one MR-scan was available for MRL treatment planning. For the MRL planning scans no monitoring of bladder filling was performed. Therefore, compared to the BT treatment plans (126 (range 35 – 240) cm^3^) where bladder filling was regulated by a urinary catheter, bladder filling in the MRL plans was variable and relatively small (64 (range 41 – 362) cm^3^), which can have negative dosimetric effects on especially the bowel. Nevertheless, in the current clinical MRL setting it is customary to keep the bladder filling relatively small, to ensure that patients can finish the treatment in the MR-Linac without an uncomfortably full bladder. Moreover, we expect limited influence on the treatment plans, as the most prominent dose-limiting organ (rectum) is not expected to be influenced by a different bladder filling.

Next to this, it was necessary to assume a continuous situation with six identical fractions, which is not true to life, as in the pelvic area movement of organs is present which induces dose delivery uncertainties [28]. Specifically for this study, which used isotoxic planning, differences in spacing between target and OARs per fraction directly impacts the dose coverage. In literature it is stated that dosimetric intra-fractional differences within BT treatment are generally small, but caution must be taken during treatment [29,30]. Van Vliet-van den Ende et al. showed that the integration of an MRI scanner into the brachytherapy suite enabled a better estimation of the delivered dose during BT treatment [16]. Ding et al. (2025) state that, based on a retrospective study, movement in the pelvic area can be corrected for using MR-guided adaptive radiotherapy [31]. Any influence on oncological outcome of uncertainties within the use of MRL due to inter- and intrafractional changes, should be evaluated in a clinical setting in the future, next to the clinical performance of MR-guided SBRT as a boost modality in general.

## Conclusion

This study demonstrates the dosimetric difference between BT and MRL as a boost modality for locally advanced cervical cancer. Although MRL treatment planning can result in similar prescribed dose values for high-volume tumours, the dose to the rectum is higher and BT and MRL dose distributions are fundamentally different. As the influence of this difference and uncertainties during treatment on oncological outcome is unknown, the use of MRL should be evaluated in clinical trials. Therefore, BT remains the modality of choice for boosting cervical cancer.

## CRediT authorship contribution statement

**Renske van Noortwijk:** Methodology, Formal analysis, Investigation, Writing – original draft, Writing – review & editing, Visualization. **Petra S. Kroon:** Conceptualization, Funding acquisition, Methodology, Validation, Supervision, Writing – review & editing. **Katelijne M. van Vliet-van den Ende:** Investigation, Resources, Writing – review & editing. **Erik H. Brondijk:** Investigation, Resources, Writing – review & editing. **Gonda G. Sikkes:** Resources, Writing – review & editing. **Alexis N.T.J. Kotte:** Resources, Writing – review & editing. **Ina M. Jürgenliemk-Schulz:** Conceptualization, Funding acquisition, Validation, Writing – review & editing. **Femke van der Leij:** Conceptualization, Funding acquisition, Validation, Writing – review & editing. **Astrid L.H.M.W. van Lier:** Conceptualization, Funding acquisition, Methodology, Validation, Supervision, Writing – review & editing.

## Funding

This work was supported by 10.13039/100011676Elekta AB (Stockholm, Sweden), grant number AGR0506. Elekta did not have any part in the design, execution or analysis of the study.

## Declaration of competing interest

The authors declare that they have no known competing financial interests or personal relationships that could have appeared to influence the work reported in this paper.
