# Supplementary material for: Dosimetric comparison between brachytherapy and MR-Linac as a boost modality for locally advanced cervical cancer
Source: Clin Transl Radiat Oncol. 2025 Dec 17;57:101098. doi: 10.1016/j.ctro.2025.101098 (PMC12795687; doi:10.1016/j.ctro.2025.101098)
Supplement: Supplementary Data 1 [file mmc1.docx]

**Supplementary material 1**

Table S.1.1: dose constraints for BT and MRL treatment, according to EMBRACE II protocol (soft and hard constraints, also described as planning aims and limits, respectively). For MRL treatment, target constraints and physical boost doses are indicated for squamous cell carcinoma. In a clinical setting, these values are increased slightly for adenocarcinoma and adenosquamous cell carcinoma.

| **BT** | | | **MRL** | | | |
| --- | --- | --- | --- | --- | --- | --- |
| **Target constraints**  **(EBRT 25x1.8 Gy + BT 4x7 Gy)**  **in Gy (EQD2 α/β=10)** | | | **Target constraints**  **including EBRT 25x1.8Gy**  **in Gy (EQD2 α/β=10)** | | | **Total physical boost dose in 6 fractions (Gy)** |
| CTV_HR_ D90% | Soft | >90  <95 | PTV-CTV_HR_ D90% | Soft | - | - |
|  | Hard | >85 |  | Hard | >85 | 25.8 |
| CTV_HR_ D98% | Soft | >75 | PTV-CTV_HR_ D98% | Soft | - | - |
|  | Hard | - |  | Hard | >75 | 31.9 |
| GTV D98% | Soft | >95 | PTV-GTV D98% | Soft | - | - |
|  | Hard | >90 |  | Hard | >90 | 34.75 |
| CTV_IR_ D90% | Soft | >60 | PTV-CTV_IR_ D90% | Soft | - | - |
|  | Hard | - |  | Hard | - | - |
| **OAR constraints** | | | **OAR constraints** | | |  |
| Bladder D2cm^3^ | Soft | <80 | Bladder D2cm^3^ | Soft | - | - |
|  | Hard | <90 |  | Hard | <90 | 29.55 |
| Rectum D2cm^3^ | Soft | <65 | Rectum D2cm^3^ | Soft | - | - |
|  | Hard | <75 |  | Hard | <75 | 23.15 |
| Sigmoid D2cm^3^ | Soft | <70 | Sigmoid D2cm^3^ | Soft | - | - |
|  | Hard | <75 |  | Hard | <75 | 23.15 |
| Bowel D2cm^3^ | Soft | <70 | Bowel D2cm^3^ | Soft | - | - |
|  | Hard | <75 |  | Hard | <75 | 23.15 |

Table S.1.2: selection criteria for group 1

|  | **Constraints (Gy EQD2 α/β = 10)** | | **# included patients** |
| --- | --- | --- | --- |
| *Criterion 1* | CTV_HR_ D90% | < 85 | 2 |
| *Criterion 2* | GTV D98% | < 90 | 5 |
| *Criterion 3* | GTV D98%  CTV_HR_ D90%  CTV_HR_ D98% | < 95  < 90  < 75 | 1 |
| *Criterion 4* | CTV_HR_ D90%  CTV_HR_ D98% | < 90  < 75 | 2 |
