# Supplementary material for: Dosimetric comparison between brachytherapy and MR-Linac as a boost modality for locally advanced cervical cancer
Source: Clin Transl Radiat Oncol. 2025 Dec 17;57:101098. doi: 10.1016/j.ctro.2025.101098 (PMC12795687; doi:10.1016/j.ctro.2025.101098)
Supplement: Supplementary Data 2 [file mmc2.docx]

**Supplementary material 2**

Table S.2: dose values in cGy corresponding to dose volumes in percentage. For both BT and MRL, dose per fraction and total boost dose are indicated.

|  |  | |  | | V150% | | V125% | | V100% | V90% | | V85% | | V75% | | V65% | | V60% | | V50% | | V25% |
| --- | --- | --- | --- | --- | --- | --- | --- | --- | --- | --- | --- | --- | --- | --- | --- | --- | --- | --- | --- | --- | --- | --- |
|  |  |  | |  | |  | |  | |  |  | |  | |  | |  | |  | |  | |
|  | BT | | dose per fraction (cGy) | | 1050 | | 875 | | 700 | 630 | | 595 | | 525 | | 455 | | 420 | | 350 | | 175 |
|  | BT | | total dose (cGy) | | 4200 | | 3500 | | 2800 | 2520 | | 2380 | | 2100 | | 1820 | | 1680 | | 1400 | | 700 |
|  |  | |  | |  | |  | |  |  | |  | |  | |  | |  | |  | |  |
| α/β = 10 |  | | total EQD2 (Gy) | | 116 | | 99 | | 84 | 79 | | 76 | | 71 | | 66 | | 64 | | 60 | | 51 |
| α/β = 3 |  | | total EQD2 (Gy) | | 157 | | 126 | | 99 | 90 | | 86 | | 78 | | 71 | | 67 | | 61 | | 50 |
|  |  | |  | |  | |  | |  |  | |  | |  | |  | |  | |  | |  |
| α/β = 10 | MRL | | total offline dose (cGy) | | 4790 | | 3955 | | 3125 | 2800 | | 2635 | | 2310 | | 1990 | | 1825 | | 1510 | | 730 |
| α/β = 10 | MRL | | dose per fraction (cGy) | | 798 | | 659 | | 521 | 467 | | 439 | | 385 | | 332 | | 304 | | 252 | | 122 |
| α/β = 3 | MRL | | total offline dose (cGy) | | 5002 | | 4150 | | 3295 | 2957 | | 2785 | | 2450 | | 2110 | | 1940 | | 1605 | | 780 |
| α/β = 3 | MRL | | dose per fraction (cGy) | | 834 | | 692 | | 549 | 493 | | 464 | | 408 | | 352 | | 323 | | 268 | | 130 |
