# Supplementary material for: Dosimetric comparison between brachytherapy and MR-Linac as a boost modality for locally advanced cervical cancer
Source: Clin Transl Radiat Oncol. 2025 Dec 17;57:101098. doi: 10.1016/j.ctro.2025.101098 (PMC12795687; doi:10.1016/j.ctro.2025.101098)
Supplement: Supplementary Data 3 [file mmc3.docx]

**Supplementary material 3**

**
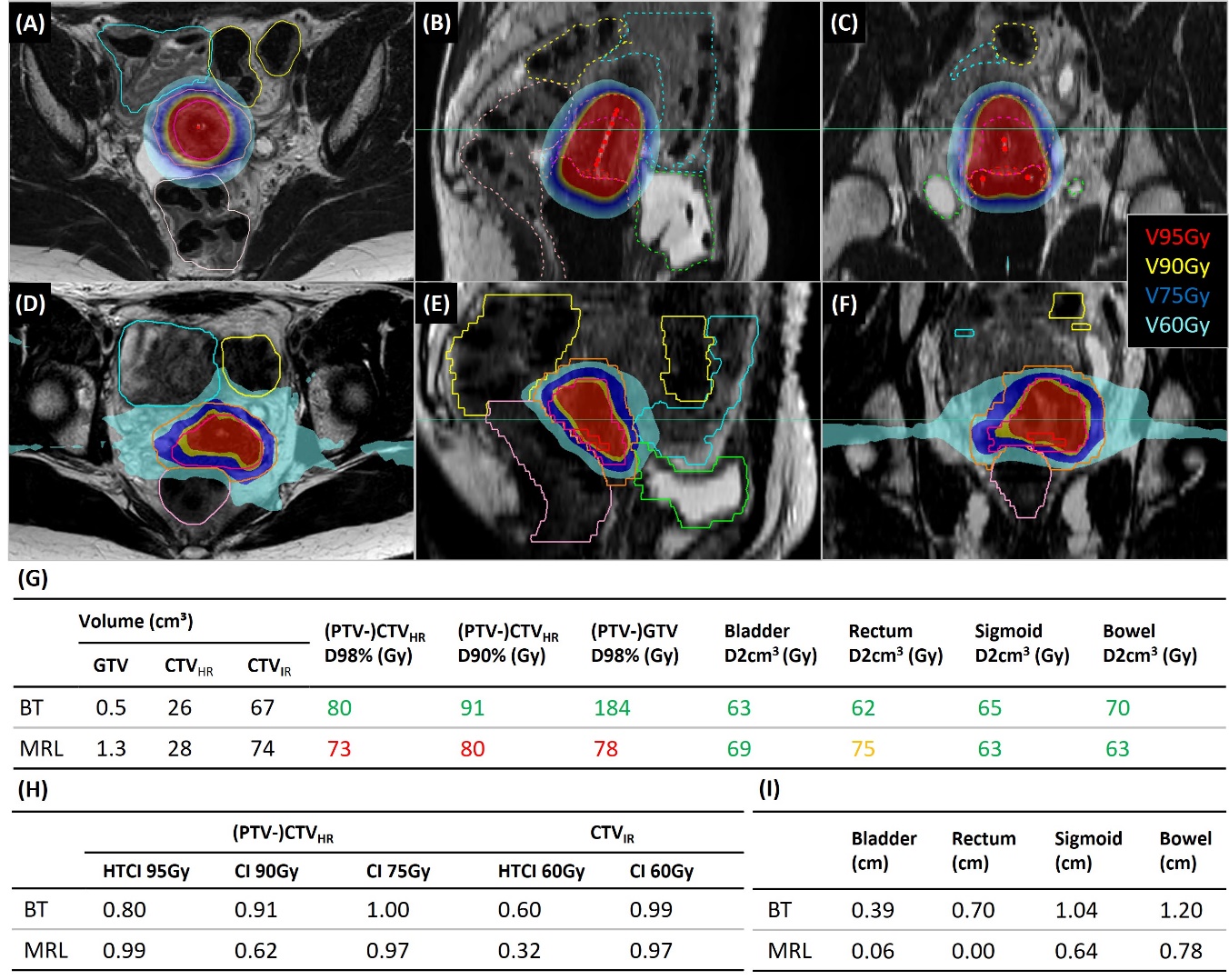
**

Figure S.3: example of a patient from group 2. Transversal, sagittal and coronal views of dose distribution for (A-C) BT treatment plans and (D-F) MRL treatment plans for the same patient. Isodoses corresponding to coloured areas are indicated: planning constraint doses, i.e. V95Gy, V75Gy and V60Gy (EQD2 α/β= 10), and dose volumes V125% and V150% (based on V100% being the volume receiving the prescription dose (7 Gy per fraction)). GTV (red), CTV_HR_ (magenta), CTV_IR_ (orange), bladder (green), rectum (light pink), bowel (blue) and sigmoid (yellow) are shown. Green lines in the sagittal and coronal view indicate the position of the corresponding transversal slide. Additionally, (G) target and OAR doses (green indicates achievement of soft constraint, orange indicates no achievement of soft constraint (hard constraint achieved if applicable), red indicates no achievement of hard constraint) (G) are included, next to (H) HTCI and CI and (I) target-OAR distances.
